# Utilization pattern of kangaroo mother care after introduction in eight selected neonatal intensive care units in China

**DOI:** 10.1186/s12887-020-02153-2

**Published:** 2020-05-29

**Authors:** Xin Liu, Zhankui Li, Xiaohui Chen, Bei Cao, Shaojie Yue, Changyi Yang, Qiongyu Liu, Chuanzhong Yang, Gengli Zhao, Qi Feng

**Affiliations:** 1 Neonatal Intensive Care Unit, Department of Pediatrics, Peking University First Hospital, Beijing, MM China; 2 Department of Neonatology, Northwest Women’s and Children’s Hospital, Shanxi, MD China; 3 Department of Neonatology, Nanjing Maternity and Child Health Care Hospital, Jiangsu, MM China; 4 Department of Neonatology, Hunan Provincial Maternal and Child Health Care Hospital, Hunan, China; 5 Department of Neonatology, Xiangya Hospital Central South University, Hunan, MD China; 6 Department of Neonatology, Fujian Provincial Maternity and Children’s Hospital, Fujian, China; 7 Department of Neonatology, Women & Children’s Health Care Hospital of Linyi, Shandong, China; 8 Department of Neonatal, Shenzhen Maternity & Child Healthcare Hospital, Shenzhen, MD China; 9 Department of Obstetrics and Gynecology, Peking University First Hospital, Beijing, MB China

**Keywords:** Preterm infants, Kangaroo mother care, Neonatal intensive care unit

## Abstract

**Background:**

Kangaroo mother care (KMC) is an evidence-based and cost-effective intervention that could prevent severe complications for preterm babies, however it has not been widely adopted in China. In this study, we aim to investigate the feasibility and parental experience of adopting KMC in a Chinese context by studying the implementation of a KMC program in eight self-selected neonatal intensive care units (NICUs).

**Methods:**

A cross-sectional study of 135 preterm infants discharged from eight NICUs in April 2018. For infants information was collected on postnatal day and corrected gestational age (GA) at KMC initiation, frequency and duration of KMC provision and whether the infant was receiving respiratory support. A nurse-administered questionnaire on parents’ knowledge and experience of KMC provision was administered to parents providing KMC.

**Results:**

One hundred thirty-five preterm infants received KMC, 21.2% of all preterm infants discharged. 65.2% of those who received KMC were below 32 weeks GA, 60.7% had a birth weight below 1500 g, and 20.7% needed respiratory support at KMC initiation. Average KMC exposure was greater in infants born at GA < 28 weeks that babies born at greater GA. 94.8% of parents that participated in the parental survey indicated that KMC was positively accepted by their family members; 60.4% of the parents claimed that KMC could relieve anxiety, 57.3% claimed it prompted more interactions with medical staff and 69.8% suggested it increased parental confidence in care for their infants.

**Conclusions:**

After advocacy, training and promotion, intermittent KMC was initiated on more immature and high-risk infants, and well-accepted by parents. We suggest continuing to promote KMC education to parents and enhancing preterm infant health.

## Background

China has sustained significant improvements in reducing child mortality, with deaths in children under the age of five falling from an estimated 18.5 per 1000 live births in 2008 to 8.6 per 1000 live births in 2018 [1]. Half of deaths in children under the age of five in China occur in the neonatal period (< 28 days) and 31% of these are due to complications of prematurity [2]. China had a preterm birth rate of 6.9 per 100 live births in 2014, equating to approximately 1.2 million preterm newborns, the second highest annual number in the world [3]. These statistics show that an intervention proven to reduce mortality and morbidity in preterm newborns if adopted across China has the potential for huge beneficial impact. Kangaroo mother care (KMC) is such an intervention.

KMC originated in Bogota, Colombia in the 1970s [4]. The World Health Organization defines KMC as “care of preterm infants carried skin to skin with the mother. Its key features include continuous and prolonged skin to skin contact between the mother and the baby, and exclusive breastfeeding (ideally) or feeding with breast milk” [5]. KMC is an evidence-based and cost-effective intervention that has been demonstrated to increase newborn survival, exclusive breastfeeding, weight gain and reduce the risk of hypothermia, apnea, and serious newborn infections such as sepsis and pneumonia [6, 7]. Evidence showed that KMC increases parent-infant bonding and improves long-term psychological and intellectual development [8, 9]. WHO recommends newborns weighing less than 2000 g receive continuous KMC when possible; and that when continuous KMC is not feasible, intermittent KMC should be provided based on evidence of decreased morbidity when compared to conventional care [10].

Before China’s Premature Birth and Preterm Infants Intervention Program started to actively promote KMC in 2014, the policy of most neonatal intensive care units (NICUs) in China was not to allow parents into the NICUs and those that permitted parental access tended to prohibit physical contact between parent and the child [11], a practice largely in place to prevent newborn infections. A few hospitals implemented KMC to varying degrees [12–14], however it was not established as part of routine care in China and there was no standardized guideline or training. Chinese doctors and nurses had few opportunities to witness or experience the provision of KMC which hindered uptake and the development of KMC implementation strategies [15]. KMC was widely perceived by clinical staff to pose an infection risk and place an additional burden on the workload of already stretched medical staff. In addition, the space available on NICUs for KMC implementation was limited and commonly cited as a major barrier for successful initiation of KMC [16].

In 2014, the Department of Maternal and Child Health of the National Health Commission of China established the Premature Birth and Preterm Infants Intervention. The Intervention Program has worked to raise awareness and promote the implementation of KMC amongst its network of 50 hospitals through the use of lectures, training in the approach, and exchange experience. Ten of these hospitals volunteered to take part in a structured pilot of KMC implementation. In 2015 the hospitals were provided with 3 days theoretical and practical training in Beijing, China. In 2016 a meeting was held between representatives of these hospitals, the Premature Birth and Preterm Infants Intervention Program and external experts to draft a KMC implementation protocol for use in the pilot. Eight of these hospitals agreed to participate in implementation research to understand the feasibility and method of adoption of KMC in the context of China’s NICUs. This would inform national KMC guidelines and create ‘centers of excellence’ to promote implementation and scale up of KMC across China. In 2017 the study design and data capture tools were developed, piloted and finalized. This cross-sectional study of KMC practice was conducted for preterm infants discharged from NICUs during the month of April 2018, it provides a snapshot of KMC implementation in participating NICU’s a couple of years after the protocol had been agreed.

## Methods

### Study design and population

This was a cross-sectional study of infants discharged from eight NICUs in April 2018. The protocol and standard operating procedures were designed by the academic study group along with international experts, with contributions from each hospital’s NICU medical and nursing staff.

The eight NICUs were self-selected from a network established under China’s Premature Birth and Preterm Infants Intervention Program, these hospitals were promoting KMC and had incorporated it into their routine practice. The hospitals from seven different provinces are located in major urban cities, two are teaching hospitals and six are specialized maternal and child-health care hospitals. All are tertiary level hospitals, the number of beds in eight NICUs rang from 22 to 60 (on average 33).

Over a two-year period, staff from the eight hospitals participated in training sessions on KMC practice and management, these trainings were delivered by international and domestic experts, in addition twice yearly workshops were held to enable staffs from participating hospitals to share their experiences of KMC implementation.

**Fig. 1:**
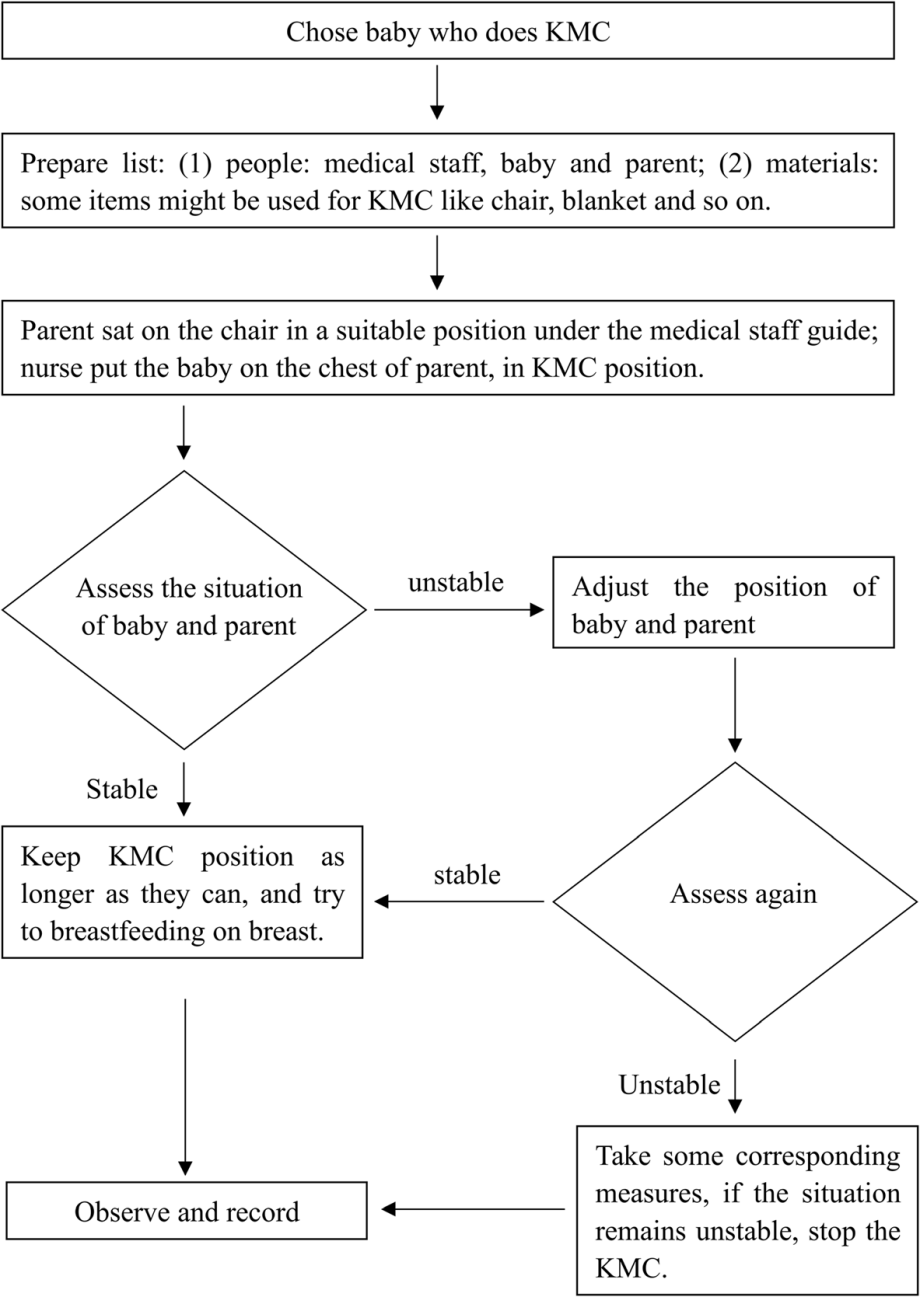
Decision support flow chart of KMC implementation

Our study population was all preterm infants (GA <  37 weeks at birth) cared for in the eight NICUs. All preterm infants were discharged in April 2018, but their date of birth varied with the earliest born in January 2018. GA was determined by both early antenatal ultrasound records and clinical examination on admission to the NICU. Hospital neonatologists decided which newborns were eligible for KMC (Fig. 1). This was based on the neonatologist’s perception of the balance of benefit and risk KMC posed for each newborn, dependent on their clinical condition. Provided a newborn’s parents were willing, intermittent KMC could then be provided. Due to the limited availability of space and staff in all eight units, it was not possible for all eligible newborns to receive KMC at the same time, therefore an appointment system was implemented, with parents booking time slots for KMC provision. Newborns receiving one or more sessions of intermittent KMC during the entire hospital stay were defined as the “KMC group”, others were defined as the “non KMC group”. Babies not considered eligible for KMC were those deemed by physicians to have very unstable vital signs, catheters in place preventing prone KMC position, and those whose parents refused to sign informed consent; NICU preterm babies considered by clinicians to be less benefited by KMC (ie, “low risk” infants) were not offered KMC given the limited available NICU space for KMC beds and the aim to provide babies who would experience greater KMC benefit if safely provided. Babies who never received KMC during the entire hospital stay for mainly these reasons were defined as the non-KMC group. In brief, the non-KMC group was largely comprised of two different groups – those considered too unstable for safe KMC; and those considered by medical staffs as “low risk” and less likely to benefit from KMC than smaller and/or sicker babies.

**Fig. 2:**
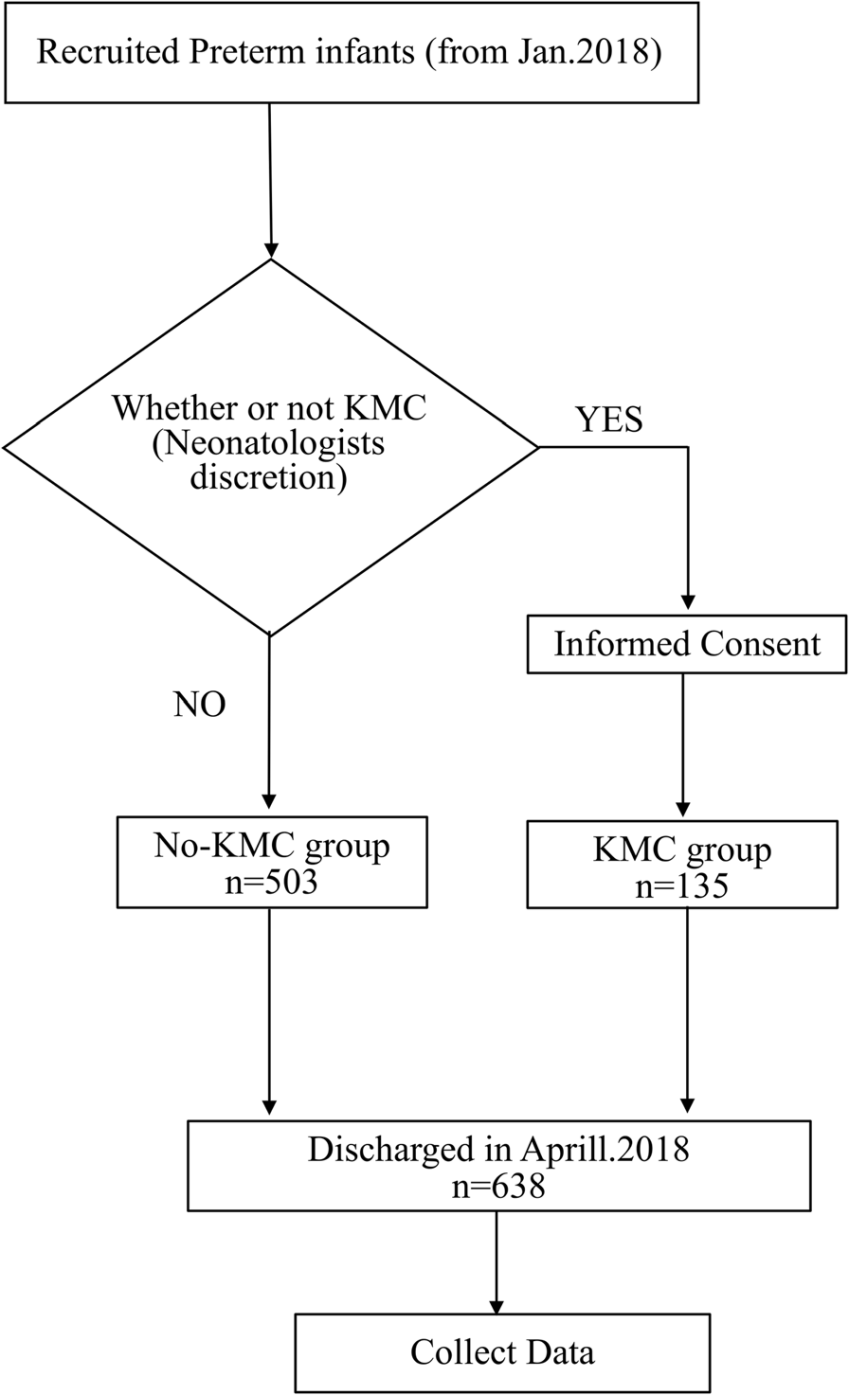
The flow diagram on the process of enrolment

KMC was practiced using a standard protocol and flow chart (Fig. 2) developed for this KMC project, mostly adapted from international KMC practice guidance [14]. Each pilot hospital took part in the development and training of the protocol and KMC flow chart.

Intermittent KMC was conducted in the NICUs on a lounge chair next to the baby’s incubator or bed. The baby was placed in the kangaroo position, skin to skin on the chest of its mother or father. Each KMC session last from 30 min to 2 hours, there’s usually 30 time slots available per day and NICU (usually from 8:00 to 20:00). The duration of each KMC session was determined by the availability of staff and space on the NICU, the infants’ condition and the parent’s availability. Likewise, the number of lounge chair next to the baby’s incubator or bed usually determine the number of slots available. NICUs and families were encouraged to practice KMC for longer duration and more sessions (> 5) during the entire hospital stay as the project progressed, based on increasing confidence of clinical staff and adaptions made in NICU environment and practice routines. During KMC, nursing staff were available to help and support the parents, they monitored the infants heart rate, respiratory rate and oxygen saturation levels. If the newborns condition deteriorated or emergency intervention was required, KMC was discontinued immediately.

### Measures and variables

General information was collected from the hospital records of all discharged preterm infants whether they received KMC or not. This included GA at birth, weight on admission and discharge, and length of hospital stay. For newborns who received KMC, each KMC session was documented by a nurse using a daily recording sheet that included information on the length of the KMC session, the infant’s age, gestational age (CGA), daily weight, vital signs and details of any respiratory support needed. The sheet also included the nurses name and the name of the parent providing KMC.

Parents providing KMC were surveyed after two or three KMC sessions. The survey questionnaire was administered by NICU nurses with the intention of collecting information on parents’ knowledge and experience of KMC provision (See supplementary appendix). Questionnaire included closed-ended questions on KMC information (source of information), KMC experience (any assistance from medical staff, perceived benefits of KMC, intention to continue KMC after discharge) and KMC preference (best time period, duration and frequency of KMC). We also included the following questions in the questionnaire: “Did you receive family member accept?”, “How did medical staff help you during KMC?”, “What are the benefits of KMC for you?”

All data was entered onto a specially designed Epidata database. A nurse from each participating NICU was identified and assumed responsibility for data entry after receiving training on data collection. Epidata data from each hospital was compiled in a central location (Peking University First Hospital) for review, reconfirmation and analysis by the authors.

Birth weight was measured using the scales available in each NICU. Respiratory support was defined as persistent oxygen via nasal cannula, invasive or non-invasive ventilation therapy. KMC exposure hours was the sum of each child’s total exposure time over the course of his/her hospital stay.

### Statistical analysis

Data analysis was performed using SPSS for Windows version 20 (IBM Inc., Chicago, IL). The test level was set at α = 0.05, and *p* <  0.05 was considered statistically significant. Outcomes were presented for the full study population and separately for the KMC and non KMC groups. Percentage and chi-square were used to analyze subject questionnaires. Statistical significance was determined by chi-square, t-test or by Wilcoxon rank sum test based on the outcome of interest.

## Results

### KMC implementation in NICUs

KMC was conducted in NICUs of all eight pilot hospitals. Table 1 shows the characteristics of preterm infants who received and did not receive KMC. Infants born at a lower gestational age (GA <  32 weeks), with a lower birth weight (BW < 1500 g), and longer stay in hospital (>14d) received more KMC.

**Table 1:** Basic characteristics of KMC and no KMC infants

	KMC group (*N* = 135)	No KMC group (*N* = 503)	T /χ^2^	*P*
Gestational age at birth (week, mean ± SD)	31.2 ± 2.3	33.9 ± 2.4	10.5	< 0.01
Gestational age < 32 weeks (%)	65.2	15.9	133.3	< 0.01
Birth weight < 1500 g (%)	60.7	15.3	117.4	< 0.01
Multiple Births^a^ (%)	38.4	37.8	0.01	0.92
Any breast feeding at discharge (%)	78.5	72.0	2.3	0.1
Length of hospital stay >14d (%)	89.6	32.3	105.9	< 0.01
Length of hospital stay >28d (%)	57.3	15.3	78.5	< 0.01
Corrected gestational age at discharge (week, mean ± SD)	34.8 ± 2.4	36.3 ± 2.1	6.0	< 0.01
Weight at discharge (g, mean ± SD)	1809.4 ± 436.1	2266.2 ± 459.5	9.2	< 0.01

*KMC* Kangaroo mother care, *SD* Standard Deviation; P, *p*-value

^a^ include twins and above, 100% minus percentage of single births

### KMC practice

Table 2 stratified infants who received KMC by GA and birth weight. 55.3% of infants below 32 weeks at birth were received KMC, and 56.9% had a birth weight of < 1500 g. More infants that were immature and with less BW received KMC.

**Table 2:** Gestational age, Birth weight distribution of KMC infants

Variable		Preterm infant discharged	Premature discharged that performed KMC, N(%)	Compared with gestational age<28 week or birth weight<1000 g	
				χ^2^	*P*
Total		638	135 (21.2%)		
Gestational age (week)	< 28	24	12 (50.0%)	–	–
	28 - < 32	135	76 (56.3%)	0.3	0.6
	32- < 34	155	35 (22.6%)	8.1	< 0.01
	34 - < 37	324	12 (3.7%)	67.6	< 0.01
*χ*^2^			171.2		
*P*			<0.01		
Birth weight (g)	< 1000	27	13 (48.1%)	–	–
	1000- < 1500	117	69 (59.0%)	1.0	0.3
	1500- < 2000	166	41 (24.7%)	6.3	0.01
	2000- < 2500	191	10 (5.2%)	41.7	< 0.01
	≥2500	137	3 (2.2%)	49.0	< 0.01
*χ*^2^			171.0		
*P*			<0.01		

*KMC* Kangaroo mother care; %, KMC premature/preterm infant discharged; χ^2^, Chi-squared test

Table 3 presents detailed information of KMC practice by infant’s GA at birth. Although babies born at a lower GA and with lower birth weight were more likely to receive KMC than premature newborns born at a greater GA and heavier birth weight, they were initiated on KMC later and once they had gained weight. Newborns with a lower gestational age at birth started KMC later compared with those whose GA ≥ 32 weeks at birth. Infants GA <  32 weeks were initiated KMC later, at day 28.0 median (IQR: 17.0,29.5) and day 23.0 (16.0,32.0) for infants with GA <  28 weeks and GA 28 - < 32 weeks respectively, compared to day 13.5 (8.8,17.3) for infants with GA ≥ 32 weeks. The weight of smaller GA infants at the time of KMC was still lower: the average weight of all KMC infants were 1809.4 ± 437.8 g, while infants GA <  28 weeks had an average weight of 1423.3 ± 226.8 g, lower than infants in other GA groups (GA 28- < 32 weeks 1768.1 ± 459.2 g(mean ± SD), GA 32- <  34 weeks 1852.8 ± 333.6 g, and GA ≥34 weeks 1995.5 ± 541.4 g). KMC duration per session (average hours of KMC received) was 1.7 ± 1.0 h on average. The duration per session and frequency of KMC did not vary by gestational age and birth weight groups, although there was a trend toward more frequent KMC in GA <  28 weeks infants.

**Table 3:** KMC initiation and practice by different gestational age group

GA (week)	KMC Number	KMC initiation			KMC practice	
		Age (d)*	CGA (week)	Weight (g)	Frequency (n)*	Duration per session (h)
Total	135	17.0 (11.0,29.0)	34.5 ± 2.0	1809.4 ± 437.8	5.0 (5.0,7.3)	1.7 ± 1.0
< 28	12	28.0 (17.0,29.5)^#^	33.9 ± 3.8	1423.3 ± 226.8	13.0 (7.5,15.0)	1.7 ± 0.3
28 ~ < 32	76	23.0 (16.0,32.0)^#^	33.7 ± 1.5^#^	1768.1 ± 459.2	5.0 (5.0,8.0)	1.8 ± 1.3
32 ~ < 34	35	14.0 (9.0,17.0)	34.9 ± 1.1	1852.8 ± 333.6	5.0 (4.0,5.0)	1.5 ± 0.7
34 ~ < 37	12	7.0 (5.0,16.5)^#^	37.2 ± 2.3^#^	1995.5 ± 541.4	5.0 (5.0,6.0)	1.5 ± 0.5
*F/χ*^2^		9.1	16.9	1.8	1.5	1.6
*P*		<0.01	<0.01	0.2	0.2	0.2

**Fig. 3:**
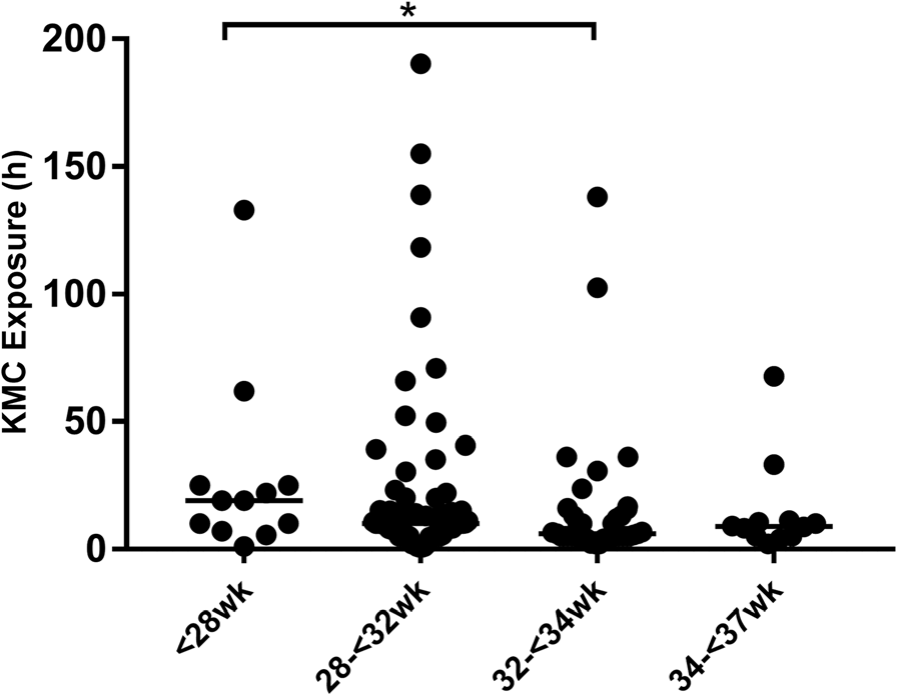
Total KMC exposure in hours per gestational age preterm infants with GA < 28wk being the longest (Median, IQR, *P* <  0.05)

*KMC* Kangaroo mother care, *GA* Gestational age, *d* Day, *CGA* Corrected gestational age, *n* Number, *h* Hour; Values are mean ± SD or median (first quartile, third quartile)(*); # denotes *p* < 0.05 while compared with 32 ~ < 34 weeks;

Figure 3 describes the distribution of accumulated KMC hours for each baby (median, quartile) differed by GA category, with significantly increased greater exposure for infants born at a lower GA (19 h for GA <  28 weeks, and 10 h, 6 h, and 8.8 h for GA 28–32 weeks, 32–34 weeks, and 34–37 weeks).

### Acceptability of KMC by parents

**Fig. 4:**
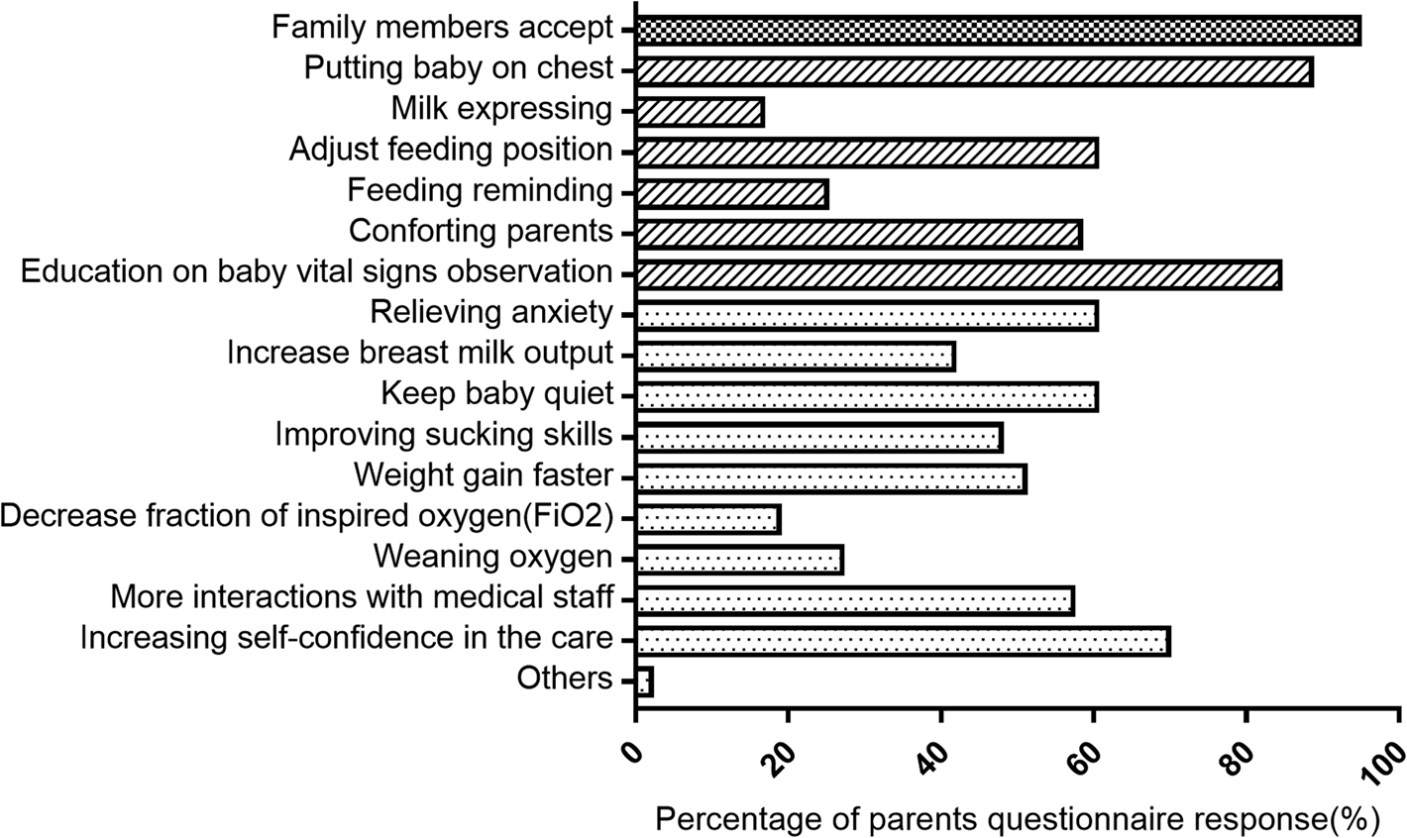
Parental experience. The bars the represent the proportion of parents who perceived that KMC had a positive effect

A total of 135 questionnaires were sent out to parents providing KMC and 135 parents responded. Parents performed KMC are invited to the survey. Questionnaire respondents could either be the father or mother, though mothers will be selected if both provided KMC. 94.8% of the parents who participated in the survey stated that KMC was positively accepted by their family members (Fig. 4). Over half of the participants indicated that KMC played a role in relieving anxiety (60.4%), increasing parental communication with doctors and nurses (57.3%), and assisted in increasing parental confidence in caring for their preterm babies (69.8%).

## Discussion

Through the analysis of cross-sectional data collected from eight NICUs in China, we found that, under current circumstances, infants born at a lower gestational age and with a lower birth weight were more likely to receive KMC than premature infants born at a greater gestational age and with a higher birth weight. First, this potentially is due to more and more tiny and sicker preterm infants to be treated in NICU and they need more extra helps besides current standard therapy. Second, medical staff’s growing confidence in KMC as a safe and beneficial intervention is important for it could be feasibly provided in NICU. The results of our program emphasis to provide KMC to small/sick babies. Preterm infants born at a GA under 28 weeks had more KMC exposure hours during hospitalization than infants born at a higher GA. KMC was well accepted by parents and was perceived as beneficial to reduce anxiety and improve communications between parents and medical staff.

Our study is one of the first to report on the implementation of KMC in China’s NICUs following its introduction in selected pilot hospitals. All eight research units included in this study are in tertiary hospitals - including both general teaching hospitals and maternal and child health care hospitals - located in major urban cities across seven different provinces in North, South, West and East China. The number of preterm infants in those NICUs are also relatively large. Our results show that intermittent KMC is both feasible and acceptable in typical NICU settings across China, and that use of KMC for relatively small and sick babies was observed. This suggests that our approach of systematic introduction, training and support for implementation may accelerate the use and scale-up of this high impact intervention across China. Despite observed differences in NICU location, bed capacity, patient population and relative differences in KMC implementation, our findings across NICUs showed largely consistent patterns of KMC use, feasibility and acceptability. We believe that combining data across these hospitals provides a valid snapshot of KMC use after introduction and establishment in China’s NICUs. Our study differs from previous studies in China that focused on very or extremely low birth weight infants admitted to NICUs in specific hospitals [17]. We looked at KMC use for all newborns admitted to eight participating NICU’s, allowing us to identify the newborns. Infants would be prioritized for KMC by neonatologists and with our geographically representative sample of hospitals our findings are more generalizable for China as a whole.

Some reports of KMC experience in other country settings indicated that KMC is commonly provided to infants with GA > 32 weeks or with a birth weight above 1500 g after becoming clinically stable [18, 19]. Our study found relatively greater KMC use for smaller and younger GA infants, including many receiving non-invasive and invasive respiratory support. Preterm infants born below 28 weeks GA had a significantly longer average KMC exposure hours compared with those born with a GA above 34 weeks. As tiny babies had more complications, longer stay in hospital, more anxious of their parents, the medical staff prefer providing some additional help. KMC is believed to be able to provide support to preterm infants by medical staffs, so in the situation of limited resource, smaller and sicker infants are more often the beneficiaries of KMC. The training for medical/nursing staff under the Premature Birth and Preterm Infants Intervention Program in fact emphasized the specific benefits for these more vulnerable babies as long as KMC was carefully used so as to ensure patient safety. In addition to our reported findings, our interviews with medical and nursing staff (not included in the current analysis) consistently found positive views of KMC benefit and safety, with common reports from staff that length of hospitalization among preterm infants was reduced following KMC introduction and increasing implementation. Our speculation appears to be in line with a recently reported national survey of NICU nurses finding increasing KMC implementation experience was associated with substantially greater nurse acceptance and perceived benefits of KMC [20].

Our analysis of the views of parents providing KMC also indicated that Chinese parents have a high level of acceptance of KMC for preterm infants. KMC was recognized as having positive influence over reducing anxiety and improving communication between parents and medical staff, which was consistent with previous studies e.g. in India [21]. It should be noted that sources of KMC information for parents in China were limited, with most parents receiving their first information on KMC from medical and nursing staff only during NICU hospitalization. While hospital-level promotion should be continued to expose more parents to the concept of KMC, additional advocacy through social media and other channels is needed to promote parental knowledge and increase “demand” for KMC in China.

Several limitations should be noted for the current study. To start with, this study is a cross-sectional study which presents the current status of KMC utilization in China’s NICUs, thus no casual inference could be made; Secondly, there might be inconsistency among hospitals regarding KMC inclusion, implementation and reporting, as KMC decision-making was largely influenced by hospital’s prior experience with KMC, and the perceived advantage and risk of KMC among individual medical staff. Nonetheless, we believe that by aggregating the data across hospitals we have the best-available evidence on KMC utilization in China’s NICUs; Thirdly, despite that one of detailed information on the discharge diagnosis which is serious infection collected, we cannot specify whether it occurred during or before the KMC sessions, which is another limitation for the current analysis.

## Conclusion

In this cross-sectional study, we analyzed and reported on the current status of intermittent KMC use in selected Chinese NICUs after prior staff exposure to advocacy and training, and the sharing of experiences among participating hospital staff. Preterm infants selected by neonatologists as eligible for KMC were found to be those who were more premature. KMC was highly accepted by parents of preterm infants. Considering safety issues like the low risk of infections, we recommend that KMC guidelines and protocols appropriate for China’s NICU settings should be developed, endorsed and implemented to enable nation-wide scale up of KMC.

## Supplementary information

**Additional file 1.** Survey questionnaire for mothers who performed KMC (filled prior to discharge).

## Data Availability

The datasets used during the current study are available from the corresponding author on reasonable request.

## Abbreviations

CGA: Corrected gestational age
GA: Gestational age
KMC: Kangaroo mother care
NICU: Neonatal intensive care unit
