# Supplementary material for: Utilization pattern of kangaroo mother care after introduction in eight selected neonatal intensive care units in China
Source: BMC Pediatr. 2020 May 29;20:260. doi: 10.1186/s12887-020-02153-2 (PMC7257127; doi:10.1186/s12887-020-02153-2)
Supplement: Supplementary file 1 — Additional file 1. Survey questionnaire for mothers who performed KMC (filled prior to discharge). [file 12887_2020_2153_MOESM1_ESM.docx]

Survey questionnaire for mothers who performed KMC (filled prior to discharge)

No. **□□□□□□**

Infant hospital record No. **□□□□□□**

Date: YYYY/MM/DD

Name:_____________

1. Did you experience any difficulty performing kangaroo mother care (KMC) during hospital stay?
2. No ②Yes (Please specify:_____________)
3. When do you prefer to perform KMC?
4. Morning ② Noon ③ Afternoon ④ Evening
5. *How long do you prefer to perform KMC everyday?*
6. < 1 hour ② 1-2 hours ③ 2-4 hours ④ 4-6 hours ⑤ 6-8 hours ⑥ > 8 hours
7. What are the reasons that will discontinue/interrupt your KMC session? (Multiple choice)
8. Time limitation ② Rest ③ Using restroom ④ Feeding ⑤ I am tired ⑥ Infant crying

⑦ Infant condition unstable ⑧ Others, please specify

5．Did you receive family support performing KMC?

① No ② Yes

6．Did you receive medical staff support performing KMC?

① No ② Yes (Continue to 6.1)

6.1 What kind of support? (Multiple choice)

① Milking ② Feeding posture adjustment ③ Feeding reminding ④ KMC posture adjustment ⑤ Consolation

⑥ Infant condition observation guidance ⑦ Others

7. What are your personal experience of KMC’s benefits? (Multiple choice)

① Relieve anxiety ② Improved lactation compared to before KMC ③ Infant is very quiet during KMC

④ Improved feeding during KMC ⑤ Better growth during KMC

⑥ Decreased fraction of oxygen support during KMC ⑦ Weaning oxygen ⑧ More interaction with doctors and nurses

⑨ Increased self-confidence in caring for the infant ⑩ Others

8．Have you heard of KMC before performing KMC in the hospital?

① No ② Yes (Continue to 8.1)

8.1 From where or whom? (Multiple choice)

① Medical staff ② People with KMC experience ③ Family and friends ④ Books and magazines

⑤ Internet and social media ⑥ Others

9．Are you going to continue KMC after you are discharged?

① Yes ② No, please specify_________________________

10．Will you recommend KMC to others?

① Yes, please specify reasons _______ ___________

________________________________________ _______ ___

② No, please specify reasons _ ___ _____________________ ___

________________________________ __________________

③ Don’t know/uncertain

11．What’s your recommendation for hospitals to improve KMC?
